# Pigment Epithelium-Derived Factor (PEDF) Receptors Are Involved in Survival of Retinal Neurons

**DOI:** 10.3390/ijms22010369

**Published:** 2020-12-31

**Authors:** Susanne Bürger, Jie Meng, Annette Zwanzig, Mike Beck, Maik Pankonin, Peter Wiedemann, Wolfram Eichler, Jan Darius Unterlauft

**Affiliations:** Department of Ophthalmology and Eye Hospital, Leipzig University, Liebigstrasse 10-14, D-04103 Leipzig, Germany; Buerger.Susanne@web.de (S.B.); Jie.Meng@medizin.uni-leipzig.de (J.M.); Annette_Zwanzig@gmx.de (A.Z.); beck.mike@gmx.net (M.B.); mp95movo@studserv.uni-leipzig.de (M.P.); Peter.Wiedemann@medizin.uni-leipzig.de (P.W.); JanDarius.Unterlauft@medizin.uni-leipzig.de (J.D.U.)

**Keywords:** retina, neuroprotection, PEDF receptor, neuron–glia interaction

## Abstract

The demise of retinal ganglion cells (RGCs) is characteristic of diseases of the retina such as glaucoma and diabetic or ischemic retinopathies. Pigment epithelium-derived factor (PEDF) is a multifunctional secreted protein that mediates neuroprotection and inhibition of angiogenesis in the retina. We have studied expression and regulation of two of several receptors for PEDF, patatin-like phospholipase 2 gene product/PEDF-R and laminin receptor (LR), in serum-starved RGC under normoxia and hypoxia and investigated their involvement in the survival of retinal neuronal cells. We show that PEDF-R and LR are co-expressed in RGC and R28 retinal precursor cells. Expression of both receptors was enhanced in the presence of complex secretions from retinal glial (Müller) cells and upregulated by VEGF and under hypoxic conditions. PEDF-R- and LR-knocked-down cells demonstrated a markedly attenuated expression of anti-apoptotic Bcl-2 family members (Bcl-2, Bcl-xL) and neuroprotective mediators (PEDF, VEGF, BDNF) suggesting that both PEDF-R and LR mediate pro-survival effects of PEDF on RGC. While this study does not provide evidence for a differential survival-promoting influence of either PEDF-R or LR, it nevertheless highlights the importance of both PEDF receptors for the viability of retinal neurons.

## 1. Introduction

Degeneration of retinal neurons is involved in sight-threatening eye diseases, such as glaucoma and ischemic retinopathies, which lead to blindness if not adequately treated. Due to the post-mitotic state of retinal ganglion cells (RGCs) and inability of these neurons to divide, their demise cannot be effectively compensated in postnatal human individuals. The worldwide prevalence of glaucoma, where progressive degeneration and programmed cell death (i.e., apoptosis) of RGC play a major pathogenic role, is estimated to exceed 110 million by the year 2040 [1]. Novel approaches for the treatment of retinal neurodegenerative diseases are urgently needed when considering currently available, ineffective treatment strategies. In particular, there are still important questions regarding mechanisms of action related to neuroprotective factors that need to be answered.

In the retina, Müller glial cells are an important source for cytoprotective factors that promote viability and survival of neurons, in particular, in response to various pathological external influences. Conditional Müller cell ablation in mice led to apoptosis of photoreceptors, thus highlighting the consequences of Müller cell dysfunction and indicating that neuron–glia interactions and glial trophic support have an important role to play for maintaining neuronal viability [2]. Among trophic Müller cell-derived mediators are, for example, pigment epithelium-derived factor (PEDF), brain-derived neurotrophic factor (BDNF), vascular endothelial growth factor-A (designated hereafter as VEGF), interleukin (IL)-6, ciliary neurotrophic factor and glial-derived neurotrophic factor [3,4,5,6,7,8,9,10,11].

PEDF, a 50-kDa monomeric glycoprotein and member of the serine protease inhibitor superfamily, regulates cellular survival, proliferation, differentiation and morphogenesis of various types of cells. Although PEDF exerts anti-angiogenic effects and thus is involved in regulating neovascular retinal disorders, its neuronal-differentiating and neuroprotective activities are important for the survival of retinal neurons under pathological conditions. The diverse functions of PEDF result from binding activities of functionally distinct modules in the PEDF molecule [12,13]. In the retina, neuroprotective actions of PEDF have been demonstrated under conditions of retinal degeneration [14] as well as oxidative stress [15], ischemia/reperfusion injury [16,17], excessive light exposition [18], glutamate excitotoxicity [19], after optic nerve transection [20], and in DBA/2J mice, an animal model of glaucoma [21]. We have demonstrated previously that increased levels of Müller-cell-derived PEDF lead to higher survival rates and longer neurite sprouts of RGC in vitro. These neuroprotective properties of PEDF were reversible by adding anti-PEDF antibodies or small interfering RNA (siRNA). Müller cell-derived PEDF promotes RGC survival, at least partly, through activation of NF-κB and STAT3 signaling pathways [3,22].

Previous studies have shown that PEDF binds to various cellular proteins such as PEDF receptor encoded by the patatin-phospholipase domain containing enzyme (PNPLA) 2 gene (PEDF-R; also referred to as adipose triglyceride lipase (ATGL)/calcium-independent phospholipase (iPL) A2ζ/human transport secretion protein TTS-2.2; [23]) and non-integrin 37/67-kDa laminin receptor/ribosomal protein SA ([24]; hereafter referred to as LR). PEDF-R is expressed in the ganglion cell layer and by retinal neuronal cells [23,25] and expression of LR in RGC has also been demonstrated [24]. However, PEDF interacts with further proteins in addition to PEDF-R and LR. These molecules involve the catalytic β subunit of F1-ATPase [26], low-density lipoprotein receptor-related protein- 6 [27], and plexin domain containing 1 and 2 (PLXDC1 and PLXDC2; [28]).

The regulation of PEDF receptors in the retina and the significance of their expression in neuronal cells are largely unknown. The purpose of this study was to determine regulation of PEDF-R and LR expression by RGC under hypoxia, which is a relevant pathological condition in retinal neurodegenerative diseases such as glaucoma or ischemic retinopathies. Furthermore, we have explored the impact of both receptors on the viability of retinal neuronal cells.

## 2. Results

### 2.1. PEDF-R and LR Are Co-Expressed in RGC and R28 Cells

To address the role of PEDF receptors in RGC, we analyzed expression of PEDF-R and LR in retinal neuronal cells in experiments mimicking conditions that contribute to the pathogenesis of retinal degenerative diseases. Experiments were carried out with growth-factor-deprived RGC and R28 cells under normoxia and hypoxia. Due to limited availability of RGC, several experiments were conducted with rat retina R28 cells only, which proved to be appropriate for the experiments in this study. In addition to PEDF-R [29] and LR [22], R28 cells express neuronal as well as glial markers [30]. Responsiveness of serum-deprived R28 cells to PEDF in live/dead assays has been reported [29,31,32]. Inducing a neuronal phenotype in R28 cells by culture of cells on a laminin-coated surface in the presence of a cell-permeable cAMP analogue [33] did not significantly interfere with effects of attenuated PEDF receptor levels on cell viability in survival assays (Supplementary Figure S1).

**Figure 1:**
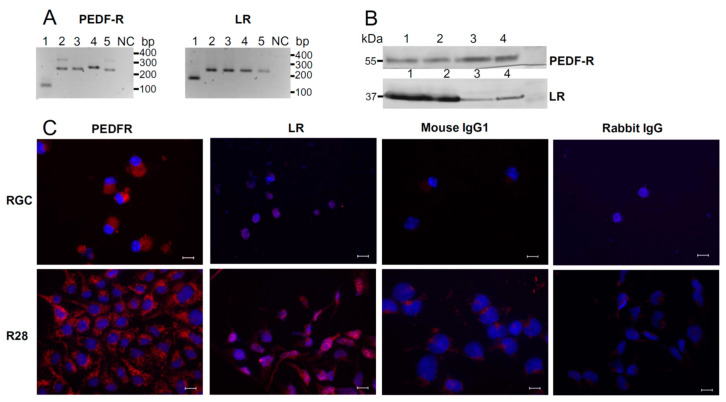
Primary retinal ganglion cells (RGC) and R28 cells express PEDF-R and laminin receptor (LR). (**A**) Total RNA was extracted from RGC (1) and R28 cells (2) and mRNA expression was examined by reverse transcription (RT)-PCR. For comparison, mRNA expression of both PEDF receptors in rat Müller (3), ARPE-19 (4) and human retinal pigment epithelium (RPE) cells (5) is demonstrated (NC: negative PCR control). (**B**) Western blot analysis revealed PEDF-R and LR expression in human RPE (1), ARPE-19 (2), rat Müller (3) and R28 cells (4). Blots were probed with antibodies against PEDF-R and LR. (**C**) Immunofluorescence of RGC and R28 cells generated by antibody labeling was detected using microscopic imaging (*red* fluorescence: PEDF-R and LR). Negative controls using non-immune mouse IgG1 or rabbit IgG were included. Cell nuclei were labeled with DAPI (*blue* fluorescence). Scale bars, 10 µm.

We first confirmed that primary RGC and/or R28 cells express PEDF-R and LR at the mRNA (Figure 1A) and protein (Figure 1B) level. Western blot analyses of lysates from R28 cells revealed that PEDF-R and LR migrated at zones (~55 and ~37 kDa, respectively), similar to those seen in separated lysates of Müller cells, retinal pigment epithelium (RPE) cells, and ARPE-19 cells (Figure 1B). Immunofluorescence microscopy confirmed that RGC and R28 cells express both PEDF receptors (Figure 1C). Taking these findings together, PEDF-R and LR are co-expressed by retinal neuronal cells raising the possibility that both of these molecules comprise functional PEDF receptors in RGC, which may account for significant PEDF-mediated pro-survival effects.

### 2.2. Complex Secretions from Müller Cells Enhance Expression of PEDF Receptors in R28 Cells under Hypoxia

**Figure 2:**
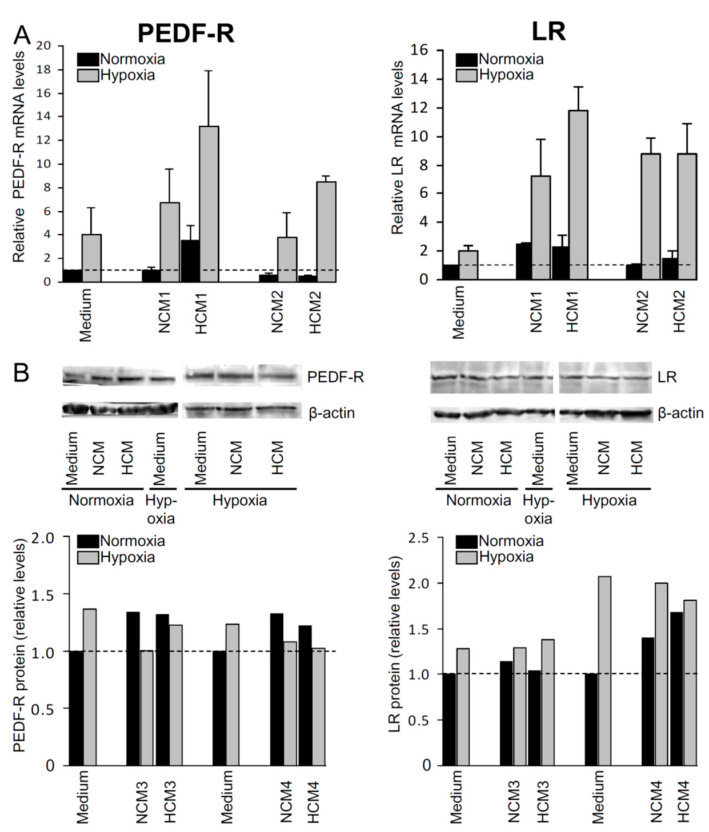
Soluble factors secreted by Müller cells enhance expression of PEDF receptors in R28 cells. R28 cells were cultured for 24 h under normoxia or hypoxia (0.2% O_2_), in the presence (NCM or HCM) or absence (“medium”) of Müller-cell conditioned media. The effect of these media on PEDF-R and LR (**A**) mRNA and (**B**) total protein expression was determined by (**A**) semi-quantitative real-time PCR and (**B**) Western blotting, respectively. Basal expression levels (*dashed* lines) relate to R28 cells cultured under normoxia in basal medium. Data in (**A**,**B**) are each derived from two independent experiments and (**A**) are expressed as means ± standard deviation and (**B**) summarize Western blotting analyses of PEDF receptor to β-actin protein expression ratios. Experiments were performed with conditioned media (1–4) derived from Müller cells of four different retinae (NCM: normoxia-conditioned media; HCM: hypoxia-conditioned media).

Retinal glial (Müller) cells are known to protect retinal neurons from a number of pathologic insults including hypoxia/ischemia [34]. We asked whether diffusible mediators secreted by Müller cells regulate the PEDF receptor level expressed by retinal neuronal cells. To this end, we cultured R28 cells in the presence or absence of Müller-cell conditioned media for 24 h, under normoxia or hypoxia (0.2% O_2_). Analyses of PEDF-R and LR expression revealed that, under hypoxic conditions, the presence of glia-conditioned media resulted in a markedly enhanced mRNA expression of both PEDF receptors in R28 cell cultures, independent of whether the media had been conditioned under normoxia (NCM) or hypoxia (HCM). However, the effect of both types of media on PEDF receptor mRNA expression was less pronounced under normoxia (Figure 2A). PEDF-R and LR upregulation induced within 24 h by glia-conditioned media was also seen at the protein level, although under hypoxia PEDF-R protein levels did not exceed normoxic levels in the presence of NCM or HCM (Figure 2B). These results suggest that the cocktail of mediators secreted by Müller cells contain (a) factor(s) appropriate to increase the levels of PEDF both receptors, thus providing permissive conditions for a PEDF-dependent mechanism through which Müller cells promote neuronal survival.

### 2.3. Regulation of PEDF-R and LR mRNA Expression in RGC and R28 Cells by VEGF and Hypoxia

**Figure 3:**
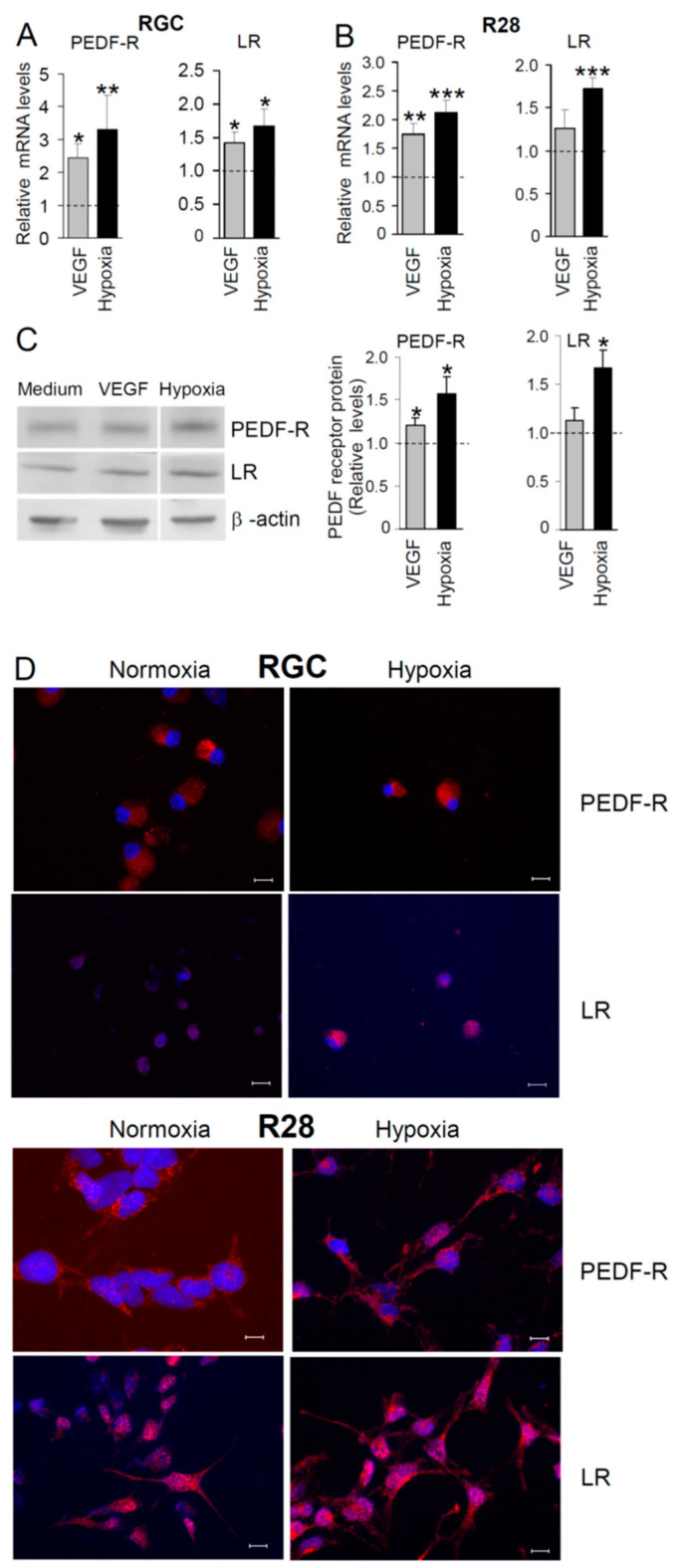
PEDF-R and LR mRNA expression in retinal neuronal cells is regulated by VEGF and hypoxia. Cells were treated with 50 ng/mL VEGF or incubated at 0.2% O_2_. At 24 h post-treatment, total RNA was prepared from (**A**) RGC or (**B**) R28 cells, and expression of PEDF receptors was analyzed by semi-quantitative real-time PCR. Shown are relative mRNA levels of PEDF-R (**A**, *n* = 8; **B**, *n* = 6–9) and LR (**A**, *n* = 6–9; **B**, *n* = 5–9). (**C**) Regulation of PEDF-R and LR proteins in R28 cells is demonstrated by a representative Western blot (*left* panel). Graphs summarizing data from independent experiments (*n* = 4–6) are shown (*right* panel). Results are expressed as relative values (fold change in PEDF-R or LR expression; * *p* < 0.05, ** *p* < 0.01; *** *p* < 0.001; means ± SEM) compared with cells of unstimulated (normoxic) control cultures (*dashed* lines). (**D**) RGC and R28 cells cultured under normoxia or hypoxia were stained with antibodies directed to PEDF-R and LR (*red* fluorescence) and immunofluorescence was detected using a fluorescent microscope. There was no staining in the control samples stained with non-immune mouse IgG1 or rabbit IgG instead of primary antibodies. Cell nuclei were labeled with DAPI (*blue* fluorescence). Scale bars, 10 µm.

The results shown in Figure 2 pointed to increased PEDF receptor levels under hypoxia; therefore, we examined PEDF-R and LR expression in RGC and R28 cells cultured for 24 h under hypoxic conditions. Given that hypoxia induces an elevated VEGF secretion by retinal glial (Müller) cells [35,36,37,38] and RGC [39], we additionally determined PEDF receptor levels in VEGF-stimulated cells. Compared to normoxic control cultures, PEDF-R mRNA expression in RGC increased 3.31-fold (*p* = 0.002) under hypoxia and 2.43-fold (*p* = 0.014) following stimulation with VEGF. Likewise, LR mRNA upregulation was induced under hypoxia in RGC 1.67-fold (*p* = 0.029) and after VEGF exposure 1.42-fold (*p* = 0.017; Figure 3A). Similar results were obtained with R28 cells (Figure 3B).

Furthermore, we performed Western blot analyses of R28 cells, which were cultured for 24 h under hypoxia or in the presence of VEGF. Shown in Figure 3C is that PEDF-R protein levels increased substantially under hypoxia (1.57-fold; *p* < 0.05, *n* = 6) or following VEGF stimulation (1.2-fold; *p* < 0.05, *n* = 6). Hypoxic conditions also elicited an increasing expression of LR protein (1.67-fold; *p* < 0.05, *n* = 4), whereas in VEGF-treated cells a tendency towards increased (1.13-fold; *n* = 5) LR levels was observed. When permeabilized RGC and R28 cells were stained for both PEDF receptors, PEDF-R was mainly localized to cytoplasma and plasma membrane of normoxic RGC and R28 cells. R28 cells demonstrated an abundant staining of PEDF-R in vesicles. Under hypoxia, PEDF-R was more strongly localized to the plasma membrane, particularly along the neurites of R28 cells. Of note, the permeabilized cells demonstrated nuclear-associated LR and, to a lesser extent, its localization in plasma membrane under normoxic and hypoxic conditions (Figure 3D).

### 2.4. Expression of Pro-Survival Factors and Neuronal Survival Are under Control of PEDF-R and LR

**Figure 4:**
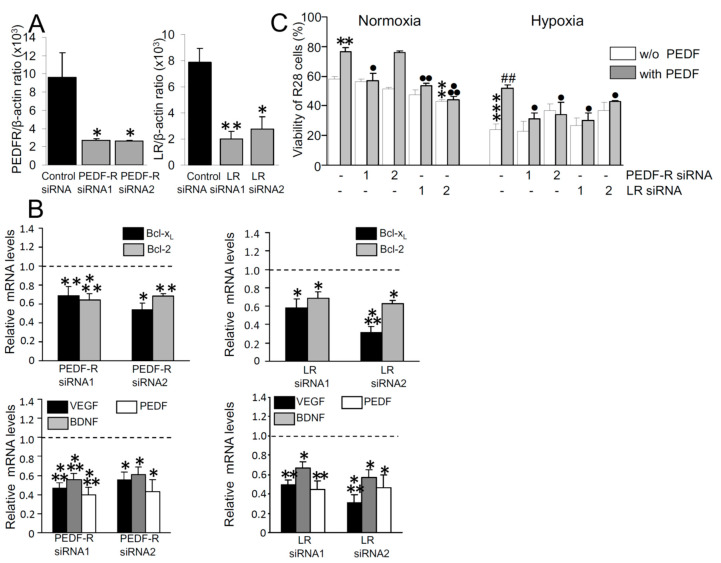
(**A**): Efficacy of PEDF-R and LR silencing induced by each of two different siRNAs in R28 cells. β-actin mRNA was used as endogenous reference transcript (means ± SEM; * *p* < 0.05, ** *p* < 0.01; *n* = 4). (**B**): PEDF-R and LR are involved in expression regulation of Bcl-2 family members (*upper* panels) and neuroprotective secreted factors (*lower* panels) in R28 cells. Cells transfected with the siRNAs indicated were cultured for 24 h and levels of Bcl-2, Bcl-x_L_, VEGF, PEDF and BDNF were analyzed by semi-quantitative real-time PCR (means ± SEM, *n* = 3–12, one sample *t* test vs. corresponding control siRNA-containing cultures (*dashed* lines); * *p* < 0.05, ** *p* < 0.01, *** *p* < 0.001). (**C**): The viability of R28 cells was determined under normoxia and hypoxia under siRNA-mediated PEDF-R and LR knockdown and control (– –, control siRNA) conditions as well as in the presence or absence of PEDF. Significant differences to normoxic (** *p* < 0.01, *** *p* < 0.001) or hypoxic (^##^ *p* < 0.01) PEDF-free control siRNA-containing, PEDF-free cultures and the significance comparing the effect of PEDF stimulation between control siRNA- and PEDF-R or LR siRNA-transfected cells (^●^ *p* < 0.05, ^●●^ *p* < 0.01, ^●●●^ *p* < 0.001) are indicated (means ± SEM; *n* = 3, one way ANOVA).

An altered PEDF receptor expression level in retinal neuronal cells may affect their susceptibility towards survival-promoted actions of PEDF. We therefore determined whether an experimentally induced loss of PEDF receptors leads to altered expression levels of genes whose products are known to counterregulate apoptosis or exert functions as neuroprotective cytokines. Knock-down of PEDF-R and LR was obtained by transfecting cells each with two different siRNAs. Compared with control siRNA, this treatment induced a 72–75% decrease in PEDF-R or LR expression in R28 cells as confirmed by using real-time PCR (Figure 4A). These experiments revealed that PEDF-R- and LR-knocked down R28 cells display significantly reduced levels of Bcl-2, Bcl-x_L_, PEDF, VEGF and BDNF mRNA compared to control cells (Figure 4B). These findings suggest that both PEDF receptor types are involved in signaling mechanisms that control the expression of cytoprotective mediators in RGC.

We further found that survival of R28 cells is significantly reduced under hypoxia (Figure 4C). When the cells were depleted of either PEDF-R or LR, almost all cell cultures did not demonstrate significantly altered survival under basal (PEDF-free) conditions, neither under normoxia nor hypoxia. However, PEDF, each under normoxia and hypoxia, induced a significant increase in cellular viability, which, however, was almost consistently mitigated in PEDF-R- and LR-knocked-down cells compared to cells treated with control siRNA (Figure 4C). These results suggest that retinal neurons, which are devoid of functional PEDF-R and LR, or are in a state of receptor downregulation, are inadequately able to transmit intracellular signals underlying PEDF-promoted cell survival.

Taking these results together, our present study suggests that PEDF-R and LR are co-expressed in retinal neuronal cells including RGC and that both molecules may have an important function for maintaining neuronal survival, in particular, under hypoxia.

## 3. Discussion

In the present study, we have investigated regulation of PEDF receptor expression in RGC under conditions that may be relevant to several retinal diseases and examined whether these receptors are involved in neuronal survival. Hypoxia/ischemia and neurotrophic/neuroprotective factor deprivation play a role in the pathogenesis of glaucoma and diabetic or ischemic retinopathies, although in diabetic retinopathy a number of additional pathogenic mechanisms may contribute to RGC death, for example, neuropathic effects induced by advanced glycation end products [40].

Our data show clear evidence that both of the receptors, PEDF-R and LR, are co-expressed in RGC and R28 cells (Figure 1). Previous experiments revealed that PEDF-R is expressed in retinal neurons, mediating the neuroprotective/neurotrophic effects of PEDF [14,29]. PEDF-R is a type-II transmembrane protein that harbors a central PEDF-binding ectodomain and, upon interaction with PEDF, undergoes an increase in its inherent calcium-independent phospholipase enzymatic activity to release fatty acids from phospholipids [23,29]. Released fatty acids such as docosahexaenoic acid act as second messengers to induce expression of neurotrophins and regeneration-associated genes [41]. However, the prototypic pro-survival signaling pathway targeted by PEDF in neurons involves activation of NF-κB, which controls transcription of anti-apoptotic genes, such as Bcl-2 and Bcl-x, and leads to upregulation of neuroprotective mediators, such as BDNF and nerve growth factor. Upregulated BDNF secretion may suppress downstream molecules in the apoptotic cascade in RGC through downregulating expression of the initiator caspase, caspase-2 [42]. By engaging the NF-κB pathway, PEDF was shown to enhance the survival of cerebellar granule cells against glutamate excitotoxicity [43]. While it has not been formally demonstrated that PEDF stimulates NF-κB activation through PEDF-R, PEDF-induced intracellular signaling involving another molecule, i.e., STAT3, is controlled by PEDF-R engagement in retinal neuronal cells [22].

LR is another receptor shown to interact with PEDF [24]. In addition to endothelial cells [24], LR is expressed in retinal neuronal and glial (Müller) cells (Figure 1). Although the significance of LR expression and contribution of LR-mediated signaling to PEDF-mediated neuroprotection need to be further explored, our results suggest that LR in retinal neuronal cells, similarly to PEDF-R, controls expression of anti-apoptotic genes such as Bcl-2 and Bcl-x_L_ and production of VEGF, PEDF and BDNF (Figure 4). Thus, it is conceivable that, through its interaction with PEDF-R and LR, PEDF is likely to access distinct pro-survival signaling pathways, which culminate in the transcriptional activation of anti-apoptotic effector molecules.

Expression and regulation of PEDF-R and LR in RGC have not yet been thoroughly investigated. We report that PEDF-R and LR levels in RGC and R28 cells are upregulated by treatment with VEGF or under hypoxia (Figure 3). Both conditions led to increased PEDF-R and LR mRNA levels as early as 4 h of exposure of R28 cells (Supplementary Figure S2). As retinal VEGF levels rise due to hypoxia [44], we considered it likely that hypoxia-induced upregulation of both PEDF receptors in RGC was at least in part attributed to upregulation of VEGF. Therefore, we have also examined whether blocking VEGF or VEGF receptors using neutralizing antibodies interferes with PEDF-R and LR upregulation. However, we did not find evidence for a significant influence of this approach on PEDF receptor expression in hypoxic R28 cells (Supplementary Figure S3). It may be concluded that elevated VEGF levels and hypoxia are likely to activate distinct signaling pathways in retinal neurons to control PEDF-R and LR upregulation, although this awaits further confirmation.

We further report that secretions from Müller glia support PEDF-R and LR expression by retinal neuronal cells (Figure 2) suggesting that Müller cells produce (a) soluble mediator(s) that induce(s) upregulation of both PEDF receptors. Since increased expression of both PEDF-R and LR mRNA was pronounced under hypoxia, we propose that, at decreasing oxygen concentrations, RGCs become increasingly conditioned for (a) Müller cell-derived secreted factor(s), which upregulate(s) PEDF-R and LR and then can more efficiently control PEDF-mediated survival-promoting actions. This is possibly a compensatory mechanism provided by Müller cells to maintain RGC viability under hypoxia/ischemia. However, cellular mechanisms regulating PEDF receptor protein expression and/or stability under hypoxia may be complex leading to attenuated or invariant hypoxic PEDF-R levels in the presence of glia-conditioned media compared to normoxia, as seen in our conditions (24 h of cell culture, cf. Figure 2B). This possibility needs to be considered in future experiments.

The identity of glial mediators accounting for upregulation of PEDF receptors in our experiments needs to be clarified. A candidate cytokine is VEGF, and upregulation of PEDF receptor levels may imply a so far unknown mechanism by which VEGF promotes neuronal survival. Earlier results from own experiments evaluating normoxic and hypoxic VEGF levels pointed to basal VEGF secretion by cultured Müller cells under normoxia and upregulation under hypoxia in vitro [38]. Hypoxia-inducible VEGF production by Müller cells was also observed in vivo [35,36]. Receptors for VEGF are constitutively expressed in the retina by cells of neural, glial and vascular origin [45,46,47], with VEGFR-2, which has been proposed to exert VEGF-mediated neuroprotection [7,8,47]. VEGF was shown to reduce the apoptosis of RGC induced by intraocular hypertension [8,48], ischemia–reperfusion injury [47] or in VEGF-overexpressing transgenic mice after optic nerve transection [7]. While VEGF may directly promote survival of RGC [8,49], studies on mice using conditional VEGF knockout [49] or RNA interference-aided VEGF inhibition in Müller cells [50,51] suggested that Müller-cell-produced VEGF may mainly control retinal neovascularization, rather than affecting RGC survival, which may be controlled through own VEGF production by the neurons [47]. However, VEGF downregulation in Müller cells is associated with photoreceptor degeneration [51], thus it remains uncertain what the precise role of Müller-cell-produced VEGF is in the context of RGC neuroprotection.

PEDF is a further Müller-cell-produced mediator, which has been shown to promote RGC survival under conditions of neurotrophic growth/survival factor deprivation or hypoxia in vitro [3,4,22]. Rescue from apoptosis under these conditions is likely to be mediated mainly by induction of anti-apoptotic Bcl-2 family members as shown previously for PEDF in immature cerebellar granule cells [41] and R28 cells [29,32,52] as well as for VEGF in R28 cells [53]. Both PEDF-R and LR may be involved in the expression control of anti-apoptotic Bcl-2 family proteins, Bcl-2 and Bcl-x_L_ (Figure 4B). This is consistent with previous findings showing that PEDF, by interacting with PEDF-R [29], upregulates Bcl-2 and blocks nuclear translocation of apoptosis-inducing factor (AIF), leading to protection of retinal neuronal cells from apoptosis [32]. LR, regarding its influence on R28 cells similar and not distinguishable to that of PEDF-R, is involved in expression regulation of Bcl-2 family members as well as of neuroprotective secretable factors, i.e., VEGF, PEDF and BDNF (Figure 4B).

Altogether, these findings suggest that upregulation of PEDF-R and LR under hypoxia sensitizes retinal neurons to interaction with PEDF, which leads to increased cell viability through induction of anti-apoptotic Bcl-2 family members, expression of secretable pro-survival factors, and suppression of apoptosis. Elucidating PEDF-R and LR function and signaling pathways regulating PEDF receptor-promoted RGC survival may be helpful in developing more efficient treatment options for retinal neurodegenerative diseases. A conceivable strategy may aim at the development of PEDF-R and/or LR agonists, which mimic neuroprotective actions of PEDF and may be of therapeutic benefit for the treatment of these diseases.

## 4. Materials and Methods

### 4.1. Animals and Cells

Animals were treated in accordance with guidelines of European Union Directive 2010/63/EU and German Law on Protection of Animals. Procedures were approved by local authorities (T 06/20, Leipzig University, Medical Faculty, approved on 13 October 2020 by Landesdirektion Sachsen). Primary rat Müller cells and mouse RGC were isolated and cultured as previously described [3,54]. Additives for cell isolation and supplements of culture media were purchased from Sigma-Aldrich (Taufkirchen, Germany) unless otherwise stated. For isolation of RGC, retinae of seven days old mice were dissected, digested for 45 min in Dulbecco’s phosphate buffered saline (D-PBS) containing papain (160 U/mL) and DNase (200 U/mL) and triturated in D-PBS containing 0.15% trypsin inhibitor and DNase (650 U/mL). The suspension was pelleted by centrifugation, resuspended in D-PBS containing 1% trypsin inhibitor, pelleted again and resuspended in D-PBS/0.02% bovine serum-albumin (BSA, fraction V). Cells were passed through a 20 µm nylon mesh, followed by isolation of RGC by immunopanning. This was accomplished by incubating the cell suspension with a rabbit anti-macrophage antibody (WAK Chemie, Steinbach, Germany; 1:75) and subsequent removal of bound contaminating microglial cells on a petri dish coated with 10 µg/mL goat anti-rabbit IgG (Dianova, Hamburg, Germany). RGCs were isolated by incubating the supernatant for 45 min on another petri dish, which had been sequentially pre-coated with goat anti-mouse IgM (Dianova) and an anti-Thy1.2 monoclonal antibody (clone F7D5; Serotec, Düsseldorf, Germany). Immunoisolated RGC were trypsinized and released cells were plated at 600 cells/mm² on glass coverslips coated with merosin (Sigma-Aldrich). Viability of cells was routinely examined under a phase contrast microscope and only viable RGC showing neurite sprouting and absence of glial contamination were selected for the experiments. Identity of Müller cells and RGC was verified using immunofluorescence staining of vimentin and neurofilament H, respectively. The immortalized retinal progenitor cell line R28 [30] was a kind gift of Dr Katharina Bell (Department of Ophthalmology, Johannes Gutenberg University, Mainz, Germany). Müller cells and R28 cells were cultured at 37 °C, 5% CO_2_, 95% air in Dulbecco’s minimal essential medium (DMEM; Thermo Fisher Scientific, Regensburg, Germany) supplemented with 10% fetal calf serum (FCS; Thermo Fisher Scientific), 100 U/mL penicillin and 100 µg/mL streptomycin. Prior to performing experiments, RGCs were cultured (37 °C, 5% CO_2_) for three days in complete neurobasal medium (Thermo Fisher) containing 0.01% BSA, 100 U/mL penicillin, 100 µg/mL streptomycin, 1 mM pyruvate, 2 mM glutamine and supplemented with 60 µg/mL *N*-acetyl-L-cysteine, 16 µg/mL putrescine, 40 ng/mL sodium selenite, 100 µg/mL BSA, 40 ng/mL triiodothyronine, 100 µg/mL holotransferrin, 250 µM dibutyryl cyclic AMP, 62 ng/mL progesterone, B-27 (1:50), 50 µM D-mannose, 10 µM forskolin, 5 µg/mL insulin, 25 ng/mL BDNF and 10 ng/mL ciliary neurotrophic factor (both from PeproTech, Hamburg, Germany). In several experiments, R28 cells were plated on laminin-coated glass slides and cultured in DMEM/10% newborn calf serum supplemented with 250 µM 8-pCPT-2′-O-Me-cyclic AMP (Sigma-Aldrich), a cell-permeable cAMP analogue. This procedure induced a neuronal phenotype in R28 cells [33]. Human RPE cells were prepared by enzymatic digestion as described previously [55]. The human RPE cell line, ARPE-19, was obtained from American Type Culture Collection (Rockville, MD, USA).

### 4.2. Hypoxia and Treatment of Cells

Experiments with RGC, R28 cells and Müller cells were performed in serum-free/growth factor-deprived media. Briefly, cells were growth arrested in serum-free media overnight, briefly washed and cultured under hypoxic (0.2% O_2_) or normoxic conditions (each at 5% CO_2_). Experiments with RGC and R28 cells were conducted in neurobasal medium and DMEM, respectively, each supplemented with 1 mg/mL BSA for 24 h. Where indicated, 50 ng/mL recombinant rat VEGF-164 (Biolegend, San Diego, CA, USA) or 100 ng/mL murine PEDF (R&D Systems, Wiesbaden, Germany) were added to RGC and/or R28 cell cultures or the cells were exposed to media (DMEM/1 mg/mL BSA) from rat Müller cells conditioned (24 h) under normoxia (NCM) or hypoxia (HCM). Cell culture experiments with neutralizing antibodies were conducted in the presence of goat anti-VEGF (R&D Systems, Cat# AF564; 5 µg/mL) and goat anti-VEGFR-2 (R&D Systems, Cat# AF357; 0.5 µg/mL). Parallel cultures treated with normal goat immunoglobulin (5 µg/mL) served as a control.

### 4.3. RNA Interference

R28 cells were plated in six-well culture plates and transfected with siRNA (Invitrogen) according to standard procedures. Briefly, siRNA (75 pmol) targeting PEDF-R (siRNA 1, sense: UCCUUAGGAGGAAUGGCCUACUGAA, antisense: UUCAGUAGGCCAUUCCUCCUAAGGA, siRNA2, sense: GCGGCAUUUCAGACAACUU[dT][dT], antisense: AAGUUGUCUGAAAUGCC GC[dT][dT]) or LR (siRNA1, sense: CUCACUCAGUGGGUCUCAU[dT][dT], antisense: AUGA GACCCACUGAGUGAG[dT][dT]; siRNA2, sense: CUCCAAUUGCUGGCCGCUU[dT][dT], antisense: AAGCGGCCAGCAAUUGGAG[dT][dT]) was complexed with Lipofectamine^®^ RNAiMAX and added to cells in antibiotic-free Opti-MEM (both from Thermo Fisher Scientific). Non-targeting control siRNA (Santa Cruz, Heidelberg, Germany, Cat# sc-37007) was used in parallel. Thirty-six hours after transfection, siRNA-transfected R28 cells were cultured in DMEM/10% FCS for 24 h and expression levels of PEDF-R, LR, Bcl-2, Bcl-x_L_, BDNF, PEDF and VEGF were determined using real-time PCR. Alternatively, siRNA-transfected cells were trypsinized 24 h after transfection, plated on glass slides and deprived of serum overnight. Viability of cells (cf. 4.5.) was determined following culture in DMEM/1 mg/mL BSA in the presence or absence of 100 ng/mL PEDF under normoxia and hypoxia for 24 h.

### 4.4. RT-PCR and Quantitation of PCR Products

Total RNA was prepared from RGC and R28 cells by standard procedures using commercially available kits (Qiagen, Hilden, Germany, or Invitek Molecular, Berlin, Germany). Reagents used for reverse transcription of RNA were from Thermo Fisher Scientific. RNA was treated with DNase I and transcribed using 200 U of Superscript IV or RevertAid™ reverse transcriptase in a 20 µL reaction containing 500 µM dNTPs, 5 mM DTT and 0.5 µg oligo(dT)_15_. Aliquots of cDNA were amplified using DreamTaq DNA Polymerase (Thermo Fisher Scientific). Primers (125 nM) used for PCR are listed in Table 1. Amplified DNA was visualized using agarose gel electrophoresis and ethidium bromide staining. In quantitative RT-PCR (qPCR) analyses, aliquots of cDNA (2 μL) were added to SsoAdvanced™ Universal SYBR^®^ Green Supermix (Bio-Rad, Munich, Germany) including 200 nM of primers. Real-time PCR was carried out at 95 °C (3 min, DNA denaturation/DNA polymerase activation), followed by 45 cycles of melting at 95 °C for 10 s and annealing/elongation at 60 °C for 50 s using a CFX Connect™ system (Bio-Rad). Mean *C*_q_ (quantification cycle) values were determined for amplification of target genes and β-actin mRNA. Relative target gene levels were obtained by calculating (1 + E)^ΔΔ*C*q^ (fold expression), with E representing amplification efficiency, Δ*C*q corresponding to each target gene expression normalized to β-actin expression (Δ*C*_q_ = *C*_q, target gene_ − *C*_q, β-actin_), and where ΔΔ*C*_q_ was calculated by subtracting experimental and control Δ*C*_q_ values.

### 4.5. Cell Viability Assays

The viability of retinal neuronal cells was assessed using calcein acetoxymethyl ester (calcein-AM; Molecular Probes, Inc., Eugene, OR, USA), a fluorogenic esterase substrate, as described previously [3]. Briefly, calcein-AM was added to culture media of RGC or R28 cells for 30 min and calcein-AM-metabolizing cells displaying green fluorescence were counted under a fluorescence microscope. To obtain the percentage of viable cells, their numbers were divided by total cell numbers obtained by counterstaining with 4′-6-diamidino-2-phenylindole (DAPI, Thermo Fisher Scientific). Ten visual fields were inspected on each coverslip at 200× magnification.

### 4.6. Analyses of PEDF Receptor Protein Expression

PEDF receptor protein levels in R28 cell lysates were analyzed by Western blotting. Side-by-side analysis of human RPE and ARPE-19 cell lysates was used to confirm antibody specificity. Cell lysates were prepared on ice, centrifuged, adjusted to 62.5 mM Tris-HCl, pH 6.8, 2% SDS, 20% glycerol, and separated on 10% polyacrylamide gels by SDS-PAGE. Blots obtained by transfer to PVDF membranes were blocked, probed with antibodies against PEDF-R (mouse anti-ATGL, Cat# sc-365278), LR (mouse anti-laminin receptor, #sc-74515; both from Santa Cruz, Heidelberg, Germany) or β-actin (Cat# 8457, Cell Signaling Technology; Frankfurt am Main, Germany), washed and exposed to goat anti-mouse immunoglobulin conjugated to alkaline phosphatase (Dianova). Blots were stained using 0.5 mg/mL nitroblue tetrazolium chloride/0.25 mg/mL 5-bromo-4-chlor-3-indolyl phosphate (both from Sigma-Aldrich) dissolved in 0.1 M Tris-HCl, pH 9.5, 0.1 M NaCl, 5 mM CaCl_2_, 5 mM MgCl_2_ and scanned using a gel documentation system (Bio-Rad, Hercules, CA). Values for PEDF receptor protein levels obtained by densitometric analysis were normalized to β-actin values and are given in relative units.

### 4.7. Immunocytochemical Staining for PEDF-R and LR

Cells plated on coverslips were fixed with 1% paraformaldehyde for 30 min on ice. After washing with TBS, cells were blocked with TBS/1% dimethyl sulfoxide/0.3% Triton X-100/3% BSA, 2 mg/mL human gamma globulin (Sigma-Aldrich) for 1 h, and incubated with antibodies directed to PEDF-R (mouse anti-ATGL, Cat# sc-365278; Santa Cruz) or LR (rabbit anti-laminin receptor, Cat# ABC934; Merck Millipore, Darmstadt, Germany) at 4 °C overnight. After washing in TBS, coverslips were incubated at room temperature with Cy3-labeled goat anti-mouse IgG or anti-rabbit IgG for 2 h. Cells were washed, counterstained with DAPI and mounted with Fluoromount-G (Thermo Fisher Scientific). Negative control stains were included using mouse IgG1 and normal rabbit IgG, respectively, at concentration similar to that of PEDF-R and LR antibodies. Images were taken using a fluorescence microscope (BZ-9000, Keyence, Osaka, Japan).

### 4.8. Data Analysis

Values are given as means ± SEM. Multiple-group comparisons were performed using standard one-way ANOVA; single comparisons were made with one sample *t*-test. Significance was accepted if *p* < 0.05.

## Figures and Tables

**Table 1:** List of oligonucleotides used for PCR amplifications.

Target Transcript (Use)	Sequence (Sense/Antisense Primers)	Amplicon Length (bp)
β-actin (qPCR)	5′-GAA ACT ACC TTC AAC TCC ATC-3′/ 5′-GAA ACT ACA TTC AAT TCC ATC-3′	170
PEDF-R (qPCR)	5′-TGT GGC CTC ATT CCT CCT AC-3′/ 5′-CAA GTT GTC TGA AAT GCC G-3′	66
PEDF-R (RT-PCR)	5′-AGA GAT GTG CAA GCA GGG-3′/ 5′-GCA CAG GCA GCA TGT TGG-3′	119 (RGC), 263 (R28), 281 (RPE cells, ARPE-19)
LR (qPCR)	5′-ACA TCA TAA ACC TGA AGA GGA C-3′/ 5′-TGG ATC TGG TTA GTG AAG GT-3′	199
LR (RT-PCR)	5′-ACATCATAAACCTGAAGAGGAC-3′/ 5′-TGGATCTGGTTAGTGAAGGT-3′	199 (RGC)
LR (RT-PCR)	5′-GGAACCCACTTAGGTGGCA-3′/ 5′-TGGATCTGGTTAGTGAAGGT-3′	275 (R28, RPE cells, ARPE-19)
VEGF (qPCR)	5′-AGA AAG CCC ATG AAG TGG TG-3′/ 5′-ACT CCA GGG CTT CAT CAT TG-3′	177
PEDF (qPCR)	5′-GAC ATG AAG CTA CAG TCC TTG T-3′/ 5′-CCC TCC TCA TTC CAC TCA AA-3′	113
BDNF (qPCR)	5′-ATGACCATCCTTTTCCTTAC-3′/ 5′-TCACGTGCTCAAAAGTGTCA-3′	205
Bcl-2 (qPCR)	5′-CTG GGA TGC CTT TGT GGA A-3′/ 5′-CAG AGA CAG CCA GGA GAA ATC A-3′	70
Bcl-x_L_ (qPCR)	5′-ATG TCT CAG AGC AAC CGG GA-3′/ 5′-CAG GAT GGG TTG CCA TTG AT-3′	170

## Data Availability

The data that support the findings of this study are available from the corresponding author upon reasonable request.

## Supplementary Materials

The following are available online at https://www.mdpi.com/1422-0067/22/1/369/s1.

## Abbreviations

ATGL	Adipose triglyceride lipase
Bcl	B-cell lymphoma
BSA	Bovine serum albumin
cAMP	Cyclic adenosine monophosphate
DAPI	4′-6-diamidino-2-phenylindole
HCM	Hypoxia-conditioned medium
LR	Laminin receptor
NCM	Normoxia-conditioned medium
PEDF	Pigment epithelium-derived factor
PEDF-R	PEDF receptor (PNPLA 2, ATGL)
PNPLA2	Patatin-phospholipase domain containing enzyme 2
qPCR	Quantitative PCR
RGC	Retinal ganglion cells
RPE	Retinal pigment epithelium
RT-PCR	Reverse transcription polymerase chain reaction
SDS-PAGE	Sodium dodecyl sulfate polyacrylamide gel electrophoresis
VEGF	Vascular endothelial growth factor
VEGFR	VEGF receptor
