# Supplementary material for: Pigment Epithelium-Derived Factor (PEDF) Receptors Are Involved in Survival of Retinal Neurons
Source: Int J Mol Sci. 2020 Dec 31;22(1):369. doi: 10.3390/ijms22010369 (PMC7795132; doi:10.3390/ijms22010369)
Supplement: Supplementary file 1 [file ijms-22-00369-s001.pdf]

Supplementary material

Pigment Epithelium-Derived Factor (PEDF) Receptors are involved in Survival of Retinal Neurons

Susanne Bürger, Jie Meng, Annette Zwanzig, Mike Beck, Maik Pankonin, Peter Wiedemann, Wolfram Eichler, Jan Darius Unterlauft

Supplementary Figures

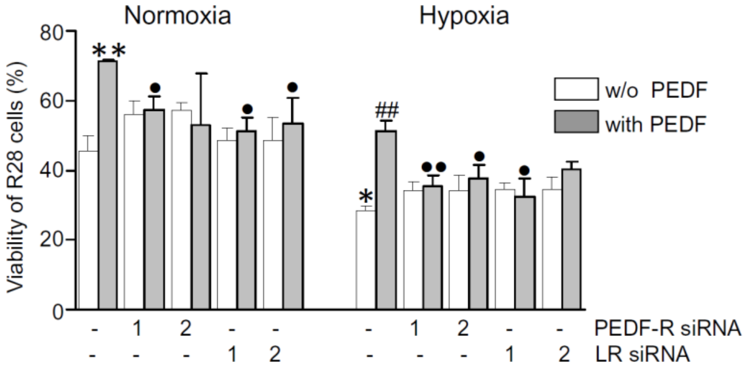

Supplementary Fig. S1. The viability of differentiated R28 cells was determined under normoxia and hypoxia, under siRNA-mediated PEDF-R and LR knockdown and control (– –, control siRNA) conditions as well as in the presence or absence of PEDF. R28 cells were cultured in laminin-coated culture plates in the presence of 250 µM 8-pCPT-2'-O-Me-cyclic AM, a cell-permeable cAMP analogue. Cell viability was determined using a calcein live/ dead assay. Significant differences to normoxic ($^{*}P < 0.05$, $^{**}P < 0.01$) or hypoxic ($^{\#\#}P < 0.01$) control siRNA-containing, PEDF-free cultures and the significance comparing the effect of PEDF stimulation between control siRNA- and PEDF-R- or LR siRNA-transfected cells ($^{\bullet}P < 0.05$, $^{\bullet\bullet}P < 0.01$) are indicated (means ± SEM; $n = 3$, one way ANOVA).

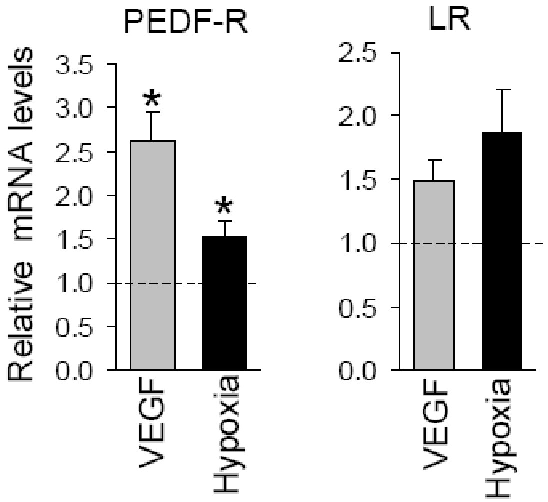

Supplementary Fig. S2. PEDF-R and LR mRNA expression in R28 cells is regulated by VEGF and hypoxia. Cells were treated with 50 ng/ml VEGF or incubated at 0.2% O_2 for 4 h. Total RNA was prepared, reverse transcribed and analyzed by qPCR. Shown are relative mRNA levels of PEDF-R and LR relative to normoxia-exposed control cultures (*dashed* lines; $n = 3 – 4$, $^{*}P < 0.05$).

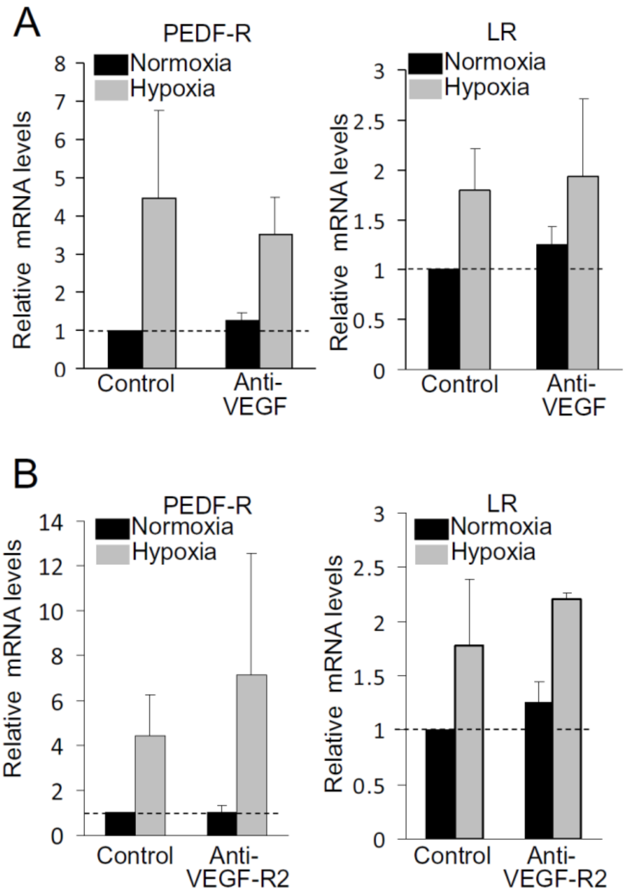

Supplementary Fig. S3. Hypoxia-induced PEDF-R and LR mRNA upregulation in R28 cells was not affected by neutralizing antibodies directed to (A) VEGF or (B) VEGF-R2. Cells were incubated at normoxia or hypoxia (0.2% O_2) in the presence of polyclonal antibodies or non-immune immunoglobulin (control). At 24 h posttreatment, total RNA was prepared and expression of genes of interest was analyzed by semi-quantitative real-time RT-PCR. Levels of PEDF-R or LR are presented relative to normoxia-exposed control cultures (*dashed* lines; $n = 3$).
